# Discovery of a Novel Dual Fungal CYP51/Human 5-Lipoxygenase Inhibitor: Implications for Anti-Fungal Therapy

**DOI:** 10.1371/journal.pone.0065928

**Published:** 2013-06-24

**Authors:** Eric K. Hoobler, Ganesha Rai, Andrew G. S. Warrilow, Steven C. Perry, Christopher J. Smyrniotis, Ajit Jadhav, Anton Simeonov, Josie E. Parker, Diane E. Kelly, David J. Maloney, S. L. Kelly, Theodore R. Holman

**Affiliations:** 1 Chemistry and Biochemistry Department, University of California Santa Cruz, Santa Cruz, California, United States of America; 2 NIH Chemical Genomics Center, National Center for Advancing Translational Sciences, Bethesda, Maryland, United States of America; 3 Institute of Life Science, College of Medicine, Swansea University, Swansea, Wales, United Kingdom; Southern Illinois University School of Medicine, United States of America

## Abstract

We report the discovery of a novel dual inhibitor targeting fungal sterol 14α-demethylase (CYP51 or Erg11) and human 5-lipoxygenase (5-LOX) with improved potency against 5-LOX due to its reduction of the iron center by its phenylenediamine core. A series of potent 5-LOX inhibitors containing a phenylenediamine core, were synthesized that exhibit nanomolar potency and >30-fold selectivity against the LOX paralogs, platelet-type 12-human lipoxygenase, reticulocyte 15-human lipoxygenase type-1, and epithelial 15-human lipoxygenase type-2, and >100-fold selectivity against ovine cyclooxygenase-1 and human cyclooxygnease-2. The phenylenediamine core was then translated into the structure of ketoconazole, a highly effective anti-fungal medication for seborrheic dermatitis, to generate a novel compound, ketaminazole. Ketaminazole was found to be a potent dual inhibitor against human 5-LOX (IC_50_ = 700 nM) and CYP51 (IC_50_ = 43 nM) *in vitro*. It was tested in whole blood and found to down-regulate LTB4 synthesis, displaying 45% inhibition at 10 µM. In addition, ketaminazole selectively inhibited yeast CYP51 relative to human CYP51 by 17-fold, which is greater selectivity than that of ketoconazole and could confer a therapeutic advantage. This novel dual anti-fungal/anti-inflammatory inhibitor could potentially have therapeutic uses against fungal infections that have an anti-inflammatory component.

## Introduction

Human 5-lipoxygenase (5-LOX) has long been considered a possible therapeutic target for inflammatory diseases. Asthma is the principle disease target, however numerous other diseases have been postulated in the literature as possible targets for 5-LOX inhibition, such as allergic rhinitis, chronic obstructive pulmonary disease, idiopathic pulmonary fibrosis, atherosclerosis, ischemia-reperfusion injury, atopic dermatitis and acne vulgaris [1]–[6]. The role of 5-LOX in the latter disease, acne vulgaris, has been shown to be related to the production of sebum in the derma [7]. 5-LOX has also been implicated in another skin disease, seborrheic dermatitis (i.e. dandruff) [8]. The involvement of 5-LOX in dandruff is because many systemic and superficial fungal infections are associated with inflammation. Ketoconazole is a widely used anti-fungal agent that is currently utilized as an active ingredient in anti-dandruff shampoo [9], [10] and previously for a wide range of fungal infections. Its mode of action is by inhibiting fungal sterol 14α-demethylase (Erg11 or CYP51) during ergosterol biosynthesis, thus retarding fungal growth [11]. However, it has been proposed that part of its effectiveness is due to its anti-inflammation activity, since it also weakly inhibits 5-LOX [12]. The anti-inflammatory activity of ketoconazole has also been seen for itraconazole, a similar anti-fungal therapeutic [13], which suggests a common theme for effective dandruff agents, dual anti-fungal/anti-inflammatory targeting. Nevertheless, the potency for ketoconazole and itraconazole against 5-LOX is poor, with IC_50_ values greater than 50 µM for both molecules, which indicates a potential for improvement in their anti-inflammatory activity [12], [13].

**Figure 1:**
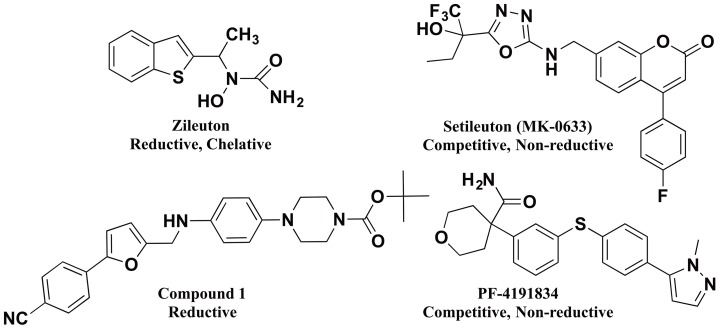
Structures of LOX inhibitors.

Numerous inhibitors for 5-LOX have been reported [14]–[17], which can be generally classified into three categories, reductive, iron ligands and competitive/mixed inhibitors [6], [18], [19] (Figure 1), however, only one compound has been approved as a drug, zileuton [20], [21]. Zileuton is a potent and selective 5-LOX inhibitor but its mode of action is unusual for a therapeutic [19], [22]. It contains an *N*-hydroxyurea moiety, which is proposed to chelate to the active enzyme's ferric ion and reduce it to the inactive ferrous ion [16], [22], [23]. In general, chelation/reduction is not considered a viable mode of inhibition for a therapeutic since metal chelation tends toward promiscuous behavior with other metalloproteins and reductive inhibitors can be chemically inactivated in the cell [16], [18], [19]. Nevertheless, zileuton has been shown to not only be selective against 5-LOX but also efficacious in the cell [20]–[22], which presents this class of inhibitors as a viable chemotype for 5-LOX inhibition. Other chelative inhibitors, such as nordihydroguaiaretic acid (NDGA) [24]–[26] are also reductive due to the facile nature of inner sphere electron reduction. NDGA contains a catechol moiety, which binds to the active site ferric ion, reducing it to the ferrous ion, with the concomitant oxidation of the catechol moiety to the semiquinone. This reactivity has previously been seen with the metalloenzyme, catechol dioxygenase, whose catechol substrate is activated to the semiquinone by the active site ferric ion for oxidation by molecular oxygen [25], [27], [28]. There is also a sub-classification of reductive inhibitors that do not chelate the active site iron. The mechanism for these inhibitors is most likely long-range electron transfer, but no direct proof has been found for this mechanism. Recent efforts by the pharmaceutical industry have focused on non-reductive inhibitors of 5-LOX (see Figure 1; setileuton and PF-4191834), however, these appear to have been discontinued during Phase II clinical trials [29], [30]. In the current publication, phenylenediamine derivatives are presented as highly selective, non-chelative, reductive inhibitors towards 5-LOX. For one derivative, the phenylenediamine core has been translated into the ketoconazole structure, generating a novel compound that demonstrates dual CYP51/5-LOX inhibitory properties. This new chemical entity, which combines anti-inflammatory and antifungal activities, is presented as a possible novel therapeutic against both the fungal and inflammatory causes of disease.

## Materials and Methods

### General methods for chemistry

All air or moisture sensitive reactions were performed under positive pressure of nitrogen with oven-dried glassware. Anhydrous solvents such as dichloromethane, *N,N*-dimethylformamide (DMF), acetonitrile, methanol and triethylamine were purchased from Sigma-Aldrich. Preparative purification was performed on a Waters semi-preparative HPLC system. The column used was a Phenomenex Luna C18 (5 micron, 30×75 mm) at a flow rate of 45 mL/min. The mobile phase consisted of acetonitrile and water (each containing 0.1% trifluoroacetic acid). A gradient of 10% to 50% acetonitrile over 8 minutes was used during the purification. Fraction collection was triggered by UV detection (220 nm). Analytical analysis was performed on an Agilent LC/MS (Agilent Technologies, Santa Clara, CA). Method 1: A 7 minute gradient of 4% to 100% Acetonitrile (containing 0.025% trifluoroacetic acid) in water (containing 0.05% trifluoroacetic acid) was used with an 8 minute run time at a flow rate of 1 mL/min. A Phenomenex Luna C18 column (3 micron, 3×75 mm) was used at a temperature of 50°C. Method 2: A 3 minute gradient of 4% to 100% Acetonitrile (containing 0.025% trifluoroacetic acid) in water (containing 0.05% trifluoroacetic acid) was used with a 4.5 minute run time at a flow rate of 1 mL/min. A Phenomenex Gemini Phenyl column (3 micron, 3×100 mm) was used at a temperature of 50°C. Purity determination was performed using an Agilent Diode Array Detector for both Method 1 and Method 2. Mass determination was performed using an Agilent 6130 mass spectrometer with electrospray ionization in the positive mode. ^1^H NMR spectra were recorded on Varian 400 MHz spectrometers. Chemical shifts are reported in ppm with undeuterated solvent (DMSO-*d*_6_ at 2.49 ppm) as internal standard for DMSO-*d*_6_ solutions. All of the analogues tested in the biological assays have purity greater than 95%, based on both analytical methods. High resolution mass spectrometry was recorded on Agilent 6210 Time-of-Flight LC/MS system. Confirmation of molecular formula was accomplished using electrospray ionization in the positive mode with the Agilent Masshunter software (version B.02).

Synthetic procedures and characterization data for compounds **1**, **2**, **6**, **8**, **12** and ketaminazole can be found in Supporting Information (File S1).

### Overexpression and purification of 5-human lipoxygenase, 12-human lipoxygenase, and the 15-human lipoxygenases

Human reticulocyte 15-lipoxygenase-1 (15-LOX-1) [31] and human platelet 12-lipoxygenase (12-LOX) [31] and human prostate epithelial 15-lipoxygenase-2 (15-LOX-2) [32] were expressed as *N*-terminally, His6-tagged proteins and purified to greater than 90% purity [33]. Human leukocyte 5-lipoxygenase was expressed as a non-tagged protein and used as a crude ammonium sulfate protein fraction, as published previously [17].

### Lipoxygenase UV-based inhibitor assay

Inhibitor potencies were evaluated through use of the standard UV-based inhibitor assay. The initial rates were determined by following the formation of the conjugated diene product at 234 nm (ε = 25,000 M^−1^ cm^−1^) with a Perkin-Elmer Lambda 40 UV/Vis spectrophotometer at one substrate concentration and varying inhibitor concentrations. All reactions were 2 mL in volume and constantly stirred using a magnetic stir bar at room temperature (23°C), with the enzyme specific buffer conditions outlined in Table 1. The substrate used for the various isozymes was arachidonic acid (AA) for 5-LOX, 12-LOX, and 15-LOX-2 and linoleic acid (LA) for 15-LOX-1, where concentrations were quantitatively determined by allowing the enzymatic reaction to go to completion. IC_50_ values were obtained by determining the initial rate at various inhibitor concentrations and plotting them against inhibitor concentration, followed by a hyperbolic saturation curve fit. The data used for the saturation curves were performed in duplicate or triplicate, depending on the quality of the data. It should be noted that all of the potent inhibitors displayed greater than 80% maximal inhibition, unless otherwise stated in the tables. Inhibitors were stored at −20°C in DMSO. As a result of screening with a semi-purified protein there was concern whether the 5-LOX concentration was approaching the inhibitor concentration for our most potent inhibitors, which would affect the Henri-Michaelis-Menten approximation. In order to investigate whether the enzyme concentration was approaching the IC_50_ value, we compared our IC_50_ values of two high potency 5-LOX inhibitors to that in the literature. Setileuton displayed an IC_50_ value of 60±6 nM, in good agreement with the literature value of 45±10 nM, and zileuton displayed an IC_50_ value of 560±80 nM, in good agreement with the literature value of 500±100 nM [2], [13]. The solvent isotope effect of the inhibitor IC_50_ was investigated utilizing the same conditions and methods as stated above. The pH of the buffered D_2_O was determined as reported previously [34].

**Table 1:** Buffer conditions for IC_50_ assays, with constant substrate concentration and varying inhibitor concentration^a^.

Enzyme	[Enyzme]	Substrate (µM)	pH	Buffer
5-LOX	crude	10 (µM) AA	7.3	25 mM HEPES, 0.3 mM CaCl_2_, 0.1 mM EDTA, 0.2 mM ATP, 0.01% Triton X-100
12-LOX	∼40 nM	10 (µM) AA	8.0	25 mM HEPES, 0.01% Triton X-100
15-LOX-1	∼20 nM	10 (µM) LA	7.5	25 mM HEPES, 0.01% Triton X-100
15-LOX-2	∼100 nM	30 (µM) AA	7.5	25 mM HEPES, 0.01% Triton X-100

a The UV-based manual inhibition data (3 replicates) were fit as described in the Materials and Methods section.

### Cyclooxygenase assay

Ovine COX-1 (Cat. No. 60100) and human COX-2 (Cat. No. 60122) were purchased from Cayman chemical. Approximately 2 µg of either COX-1 or COX-2 were added to buffer containing 100 µM AA, 0.1 M Tris-HCl buffer (pH 8.0), 5 mM EDTA, 2 mM phenol and 1 µM hematin at 37°C. Data was collected using a Hansatech DW1 oxygen electrode chamber, as described before [35]. Inhibitor or vehicle were mixed with the respective COX in buffer within the electrode cell, the reaction was initiated by the addition of AA, followed by monitoring of rate of oxygen consumption. Ibuprofen, aspirin and indomethacin, and the carrier solvent, DMSO, were used as positive and negative controls, respectively.

### Human blood LTB4 inhibition assay

Whole human blood was obtained from healthy volunteers from within the Student Health Center. These studies were approved by the UCSC Institutional Review Board (IRB) and informed consent was obtained from all donors before blood draw. The whole blood was dispensed in 150 µL samples followed by addition of inhibitor or control (vehicle, DMSO), and incubated for 15 min at 37°C. The mixture was then stimulated by introduction of the calcium ionophore, A23817, (freshly diluted from a 50 mM DMSO stock to 1.5 mM in Hanks balanced salt solution), and incubated for 30 min at 37°C. Samples were then centrifuged at 1,500 rpm (300 g) for 10 min at 4°C and the supernatant diluted between 20 and 50-fold (batch dependent) for LTB4 detection, using an ELISA detection kit (Cayman Chemicals Inc.). Inhibitors were added at 10 µM concentrations [36]–[38] and the IC_50_ values were generated using a one point IC_50_ estimation equation.

### Pseudoperoxidase activity assay

The reductive properties of the inhibitors were determined by monitoring the pseudoperoxidase activity of lipoxygenase in the presence of the inhibitor and 13-(S)-hydroperoxyoctadecadienoic acid (13-HPODE). Activity is monitored by direct measurement of the product degradation following the decrease of absorbance at 234 nm using a Perkin-Elmer Lambda 40 UV/Vis spectrometer (50 mM Sodium Phosphate (pH 7.4), 0.3 mM CaCl_2_, 0.1 mM EDTA, 0.01% Triton X100, 10 µM 13-HPODE). All reactions were performed in 2 mL of buffer and constantly stirred with a rotating stir bar (23°C). Reaction was initiated by addition of 10 µM inhibitor (a 1 to 1 ratio to 13-HPODE), and a positive result for activity reflected a loss of greater than 40% of product absorption at 234 nm. The control inhibitors for this assay were zileuton, a known reductive inhibitor [18], and setileuton, a competitive inhibitor [29].

### Inhibitor modeling

Grid generation and flexible ligand docking were performed using Glide, while energy minimization and ligand preparation of inhibitors was done with LigPrep. LigPrep and Glide are both products of Schrodinger, Inc., and utilize energy functions to generate and rank models of ligand 3D structures and ligand-protein interactions, respectively. The crystal structure of stable human 5-lipoxygenase (PDB ID: 3O8Y) was used to generate a Glide grid in which to carry out docking algorithms with our inhibitors. This structure contains several point mutations that remove destabilizing sequences, but since none of these are located at the active site of the enzyme, it is reasonable to assume the mutant structure is an accurate model of the wild-type active site. Positional constraints at the catalytic iron and at hydrophobic pockets within the active site were prepared and utilized intermittently during different docking calculations. Poses generated from ligand docking were ranked according to their GlideScores.

### CYP51 protein studies

*C. albicans* CYP51 (CaCYP51) and *Homo sapiens* CYP51 (HsCYP51) proteins were expressed in *E. coli* using the pCWori^+^ vector, isolated and purified as previously described [39], [40] to over 90% purity. Native cytochrome P450 concentrations were determined by reduced carbon monoxide difference spectra [41], based on an extinction coefficient of 91 mM^−1^ cm^−1^ [42], [43]. Binding of azole antifungal agents to 5 µM CaCYP51 and 5 µM HsCYP51 were performed as previously described [39], [44], using 0.25 and 0.5 mg mL^−1^ stock solutions of ketoconazole and ketaminazole in DMSO. Azole antifungal agents were progressively titrated against the CYP51 protein in 0.1 M Tris-HCl (pH 8.1) and 25% (wt/vol) glycerol, with the spectral difference determined after each incremental addition of azole. The dissociation constant (*K_d_*) of the enzyme-azole complex was determined by nonlinear regression (Levenberg-Marquardt algorithm) of Δ*A*_peak-trough_ against azole concentration using a rearrangement of the Morrison equation [45] and fitted by the computer program ProFit 6.1.12 (QuantumSoft, Zurich, Switzerland).

IC_50_ determinations were performed using the CYP51 reconstitution assay system previously described [46], [47], containing 1 µM CaCYP51 or 0.3 µM HsCYP51, 2 µM human cytochrome P450 reductase, 50 µM lanosterol, 50 µM dilaurylphosphatidylcholine, 4.5% (wt/vol) 2-hydroxypropyl-β-cyclodextrin, 0.4 mg mL^−1^ isocitrate dehydrogenase, 25 mM trisodium isocitrate, 50 mM NaCl, 5 mM MgCl_2_ and 40 mM MOPS (pH∼7.2). Azole antifungal agents were added in 5 µL DMSO followed by incubation for 5 minutes at 37°C prior to assay initiation with 4 mM β-NADPHNa_4_, with shaking for a further 10 minutes at 37°C. Sterol metabolites were recovered by extraction with ethyl acetate followed by derivatization with *N*,*O*-bis(trimethylsilyl)trifluoroacetamide and tetramethylsilane prior to analysis by gas chromatography mass spectrometry [48]. The term IC_50_ is defined as the inhibitor concentration required for a 50% inhibition of the CYP51 reaction under the stated assay conditions.

## Results and Discussion

**Figure 2:**
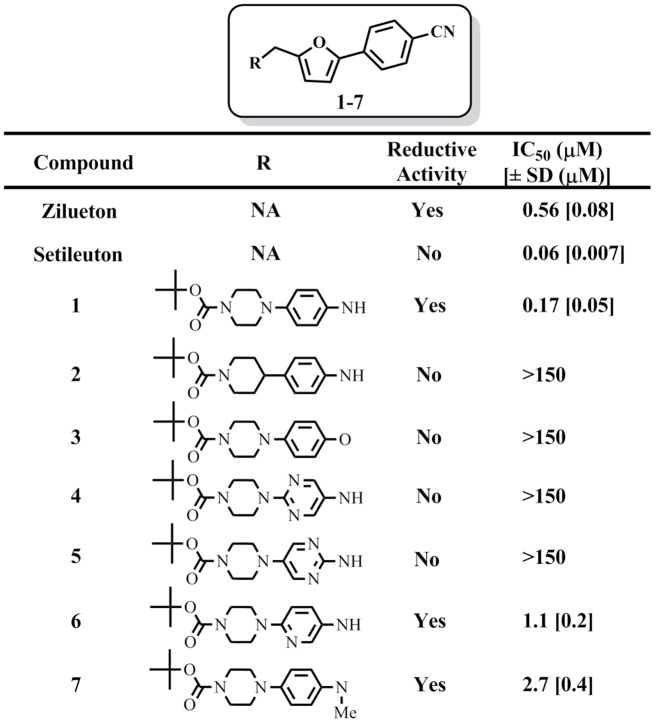
Representative analogues evaluated for pseudoperoxidase activity and IC_50_ potency (µM), with errors in brackets. The UV-based manual inhibition data (3 replicates) were fit as described in the Materials and Methods section.

Our laboratories have previously utilized a high-throughput screen to discover inhibitors against 12-LOX [63] and 15-LOX-1 [35]. In the process of screening the 15-LOX-1 “hits”, we serendipitously discovered a novel 5-LOX inhibitor with a phenylenediamine core moiety, compound **1**. Due to its chemical nature, the mode of inhibition was postulated to be due to reduction of the active site ferric atom. As mentioned in the introduction, reductive inhibition of lipoxygenase is a very effective mode of action, with many reductive inhibitors having sub-micromolar IC_50_ values [5], [6], [16], [19]. This fact is indicative of both the ease with which the active site ferric can be reduced and the importance of the oxidation state of the iron. With this in mind, the phenylenediamine parent compound (**1**) was modified to change its reduction potential (Figure 2) [49]. Modifications of the phenylenediamine core, such as atom substitutions of the nitrogens with carbon or oxygen (**2** and **3**, respectively), or the insertion of two additional nitrogen atoms into the core phenyl group (**4**, **5**), resulted in complete loss of inhibition. Interestingly, substitution of only one nitrogen into the core phenyl ring (**6**) did not lower potency dramatically, nor did methylation of the nitrogen (**7**).

The pseudoperoxidase assay, was subsequently conducted with these inhibitors to establish their reductive activity against 5-LOX (Figure 2). From these data, it was demonstrated that the pseudoperoxidase activity paralleled their inhibitor potency, consistent with changes in the reductive potential of the inhibitors. Similar alterations of the core phenylenediamine structure were previously used in a similar manner to determine the relationship between potency and reductive properties [50]–[53]. For comparison, zileuton and setileuton were screened as positive controls, with zileuton being reductive and setileuton being non-reductive in their mechanism of inhibition.

**Figure 3:**
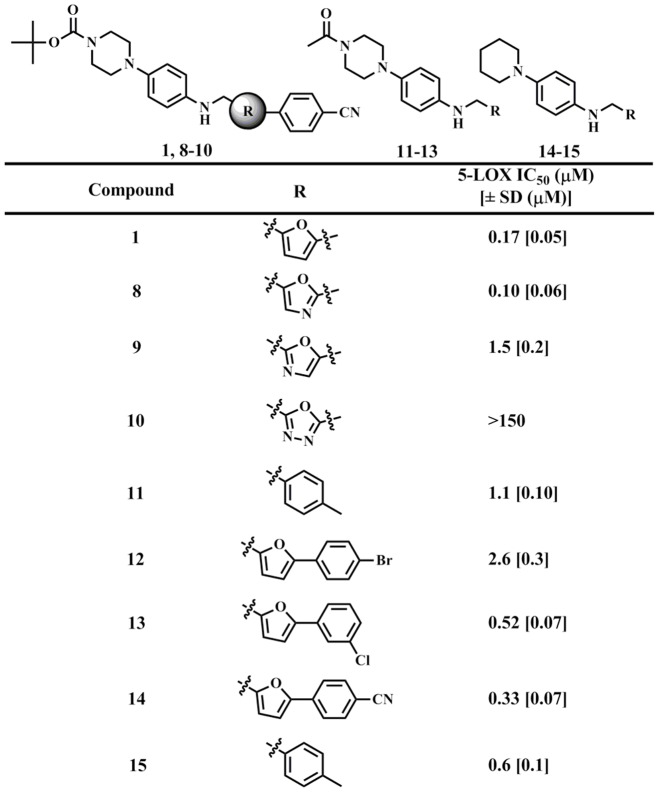
5-LOX IC_50_ values of representative analogues (µM), with errors in brackets. The UV-based manual inhibition data (3 replicates) were fit as described in the Materials and Methods section.

Interpreting IC_50_ values for reductive inhibitors is challenging because their relative potency is dependent on a combination of both their reactivity with the active site iron and their binding affinity. The binding affinity was therefore investigated by changing the substituents on either side of the phenylenediamine core. As seen in Figure 3, the chemotype core tolerated a large range of modifications, such as changing the steric bulk on either side of the phenylenediamine core. Only one modification in this small set of compounds showed a greater than 10-fold decrease in potency, **10**, which was surprising given the activity of related oxazoles **8** and **9**. The lack of dependence between inhibitor potency and inhibitor structure suggests that the active site can accommodate a variety of inhibitor shapes and sizes. These findings are consistent with the large size of the 5-LOX active site [54] and the re-occurrence of large 5-LOX inhibitors discovered [6], [16], [19].

Selectivity of the inhibitor chemotype was evaluated by screening a variety of LOX isozymes with a small subset of compounds (Table 2). Strong selectivity was displayed against 5-LOX relative to the other isozymes, with selectivity ratios ranging from 80-fold for 12-LOX, 75-fold for 15-LOX-1, and 30-fold for 15-LOX-2, for the least selective analogues (Table 2). The chemotype also displayed strong selectivity when assayed against cyclooxygenase (COX), with a 140-fold selectivity versus COX-1, and a 240-fold selectivity versus COX-2. These combined results indicate this chemotype has a strong preference/selectivity against 5-LOX versus other AA processing enzymes. As controls, setileuton and zileuton were utilized as selective inhibitors of 5-LOX [20], [21], [29].

**Table 2:** Selectivity profile of representative analogues (µM), with errors in parentheses^a^.

Compound	5-LOX	12-LOX	15-LOX-1	15-LOX-2	COX-1	COX-2
**1**	0.17 (0.05)	>150	>150	>150	>50	>150
**13**	0.52 (0.07)	>50	>50	>50	>150	>150
**14**	0.33 (0.07)	>150	>25	10	>150	N/D^b^
**15**	0.60 (0.1)	>50	>150	>50	N/D	N/D
Setileuton	0.060 (0.007)	>150	>150	>150	>150	>50
Zileuton	0.56 (0.08)	>150	>50	>150	>150	N/D

a The UV-based manual inhibition data (3 replicates) were fit as described in the Materials and Methods section.

b N/D = Not determined.

A few inhibitors were then tested for efficacy in whole human blood, which is known to express 5-LOX upon activation by an ionophore. **1** and **13** displayed roughly 50% inhibition at 10 µM drug dosing in the whole blood, while the positive control, setileuton, was found to inhibit 100% at 10 µM (Table 3). Compound **15** was also tested, but the potency was shown to be weak, with less than 10% inhibition at 10 µM (Table 3). The cellular enzyme inhibition for **1**, **13** and setileuton are diminished relative to the isolated-enzyme inhibitor values (Figure 2). This result, along with other analogues failing to display high potency, could indicate poor permeability, plasma protein binding, non-specific interactions or metabolism of the inhibitors by the cell.

**Table 3:** Whole human blood activity profile of representative analogues^a^.

Compound	Inhibition (%)
Setileuton	100 (11)
Cicloproxin	34 (2)
Ketaminazole (**16**)	45 (10)
Ketoconazole	25 (3)
**1**	65 (14)
**13**	54 (11)
**15**	8 (1)

a The ELISA absorption-based inhibition data (3 replicates) were fit as described in the Materials and Methods section. Compounds were assayed at 10 µM.

**Figure 4:**
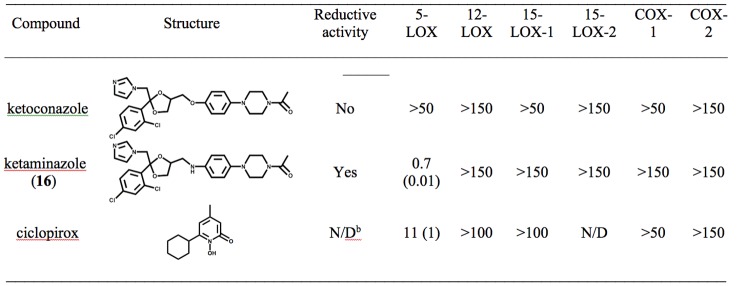
IC_50_ values of dual anti-fungal, anti-inflammatory inhibitors (µM), with error in parentheses. The UV-based manual inhibition data (3 replicates) were fit as described in the Materials and Methods section. N/D = Not determined.

The determination that the reductive phenylenediamine core was the key potency component and that the addition of large functionalities to either side of the phenylenediamine core was well tolerated led us to consider the similarity between the phenylenediamine chemotype and ketoconazole (Figure 4). Ketoconazole is a CYP51 inhibitor with an azole moiety that targets the active site heme and is a potent antifungal medication [8], [9]. In addition, ketoconazole was previously determined to inhibit 5-LOX and have anti-inflammatory properties, although weakly [12]. Considering the similarity of ketoconazole to our chemotype, we hypothesized that by adding the phenylenediamine core to ketoconazole, we could improve its 5-LOX potency by making it a reductive inhibitor and thus increasing its anti-inflammatory properties. We subsequently modified the structure of ketoconazole to include a phenylenediamine core to generate a novel compound, ketaminazole (**16**) and found that its potency against 5-LOX increased over 70-fold compared to ketoconazole (Figure 4) and that it was a reductive inhibitor, as seen by its activity in the pseudoperoxidase assay (Figure 4). The selectivity of the ketaminazole (**16**) was also investigated and found to preferentially inhibit 5-LOX over 100 times better than that of 12-LOX, 15-LOX-1, 15-LOX-2, COX-1 and COX-2 (Figure 4). This is most likely due to the large active site of 5-LOX compared to the other human LOX isozymes. Ketaminazole (**16**) was also tested in whole human blood and shown to display cellular activity. Like the smaller phenylenediamine inhibitors (**1**, **13** and **15**), ketaminazole's cellular potency is lower relative to its *in vitro* potency, displaying an approximately 20-fold reduction (Table 3). The magnitude of the potency in whole blood is not consistent between all the phenylenediamine inhibitors tested. This indicates that the structural differences between the phenylenediamine inhibitors have an effect on their cellular potency, supporting the hypothesis that cellular factors, other than the phenylenediamine core, are important. Gratifyingly, ketaminazole (**16**) displayed a better potency against 5-LOX in whole blood relative to ketoconazole, however, the magnitude of this difference was not as great as their *in vitro* difference. This is surprising since their only structural difference is the substitution of an amine for the ether linkage. It could be that the polarity change of the inhibitors changes their cellular uptake or that the reductive state of the ketaminazole is being compromised in the cell. Further cellular studies are required to probe these hypotheses further.

**Figure 5:**
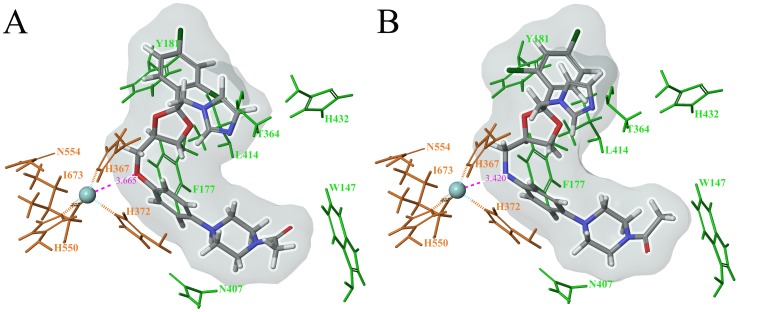
Docking ketoconazole (A) and ketaminazole (B) to the crystal structure of the Stable-5-LOX (PDB ID: 3O8Y). Glide docking scores and poses were similar to other high-ranking docked inhibitors.

In addition to kinetic data, the importance of the phenylenediamine core for reductive inhibition was further supported using computational methods. Molecular modeling of possible inhibitor binding modes within the active site was initiated by deprotonation of the amine groups at the phenylenediamine core and energy minimization of the compounds with LigPrep [55], [56]. The inhibitors listed in of the Figures/Tables above were then docked against the crystal structure of modified protein, Stable-5-LOX (3O8Y), using Glide's “XP” (extra-precision) mode [55], [56]. Different trials, with varying Van der Waals scaling factors and alternating positional or hydrophobic constraints linking the inhibitor to the active site, resulted in the occurrence of high-ranking binding poses depicting the deprotonated amine nitrogen within 10 angstroms of the catalytic iron for several inhibitors. The docking results of these inhibitors support the hypothesis that the reduction of the ferric iron could be caused by the phenylenediamine core, either through an inner sphere (direct coordination to the iron) or outer sphere (through space) mechanism [57]. Docking of the larger inhibitors, ketoconazole (Figure 5a) and ketaminazole (**16**) (Figure 5b), generated poses with similar Glide docking scores to the other inhibitors studied, suggesting a comparable binding mode despite the differences in IC_50_ values. In several high-ranking binding poses, the amine/ester core of ketaminazole (**16**) was observed to be within 5 angstroms of the catalytic iron (Figure 5b), supportive of the hypothesis that the phenylenediamine core reduces the active site iron.

The docking poses of the phenylenediamine inhibitors suggest that their amine moieties could be possible conduits of iron reduction, through space via an outer sphere mechanism [57]. However, the docking poses also suggest the active site iron-hydroxide moiety could possibly abstract a hydrogen atom from the amine by an inner sphere mechanism, as is seen in the natural mechanism of LOX with its fatty acid substrate [58]. To test this hypothesis, **13** was incubated in D_2_O buffer, to deuterate the phenylenediamine core amine, and its IC_50_ value compared to the protonated amine in H_2_O. A 2.4-fold increase in the IC_50_ for **13** was observed in D_2_O, which is well below the kinetic isotope effect expected for hydrogen atom abstraction [57], suggestive of a proton independent outer sphere reductive mechanism. To further verify this proton-independent reductive mechanism, **1** and **7** (containing the protonated and methylated amine, respectively) were also investigated and both were shown to have similar increases in IC_50_ values in D_2_O relative to H_2_O, suggesting the effect does not involve the amine proton.

**Figure 6:**
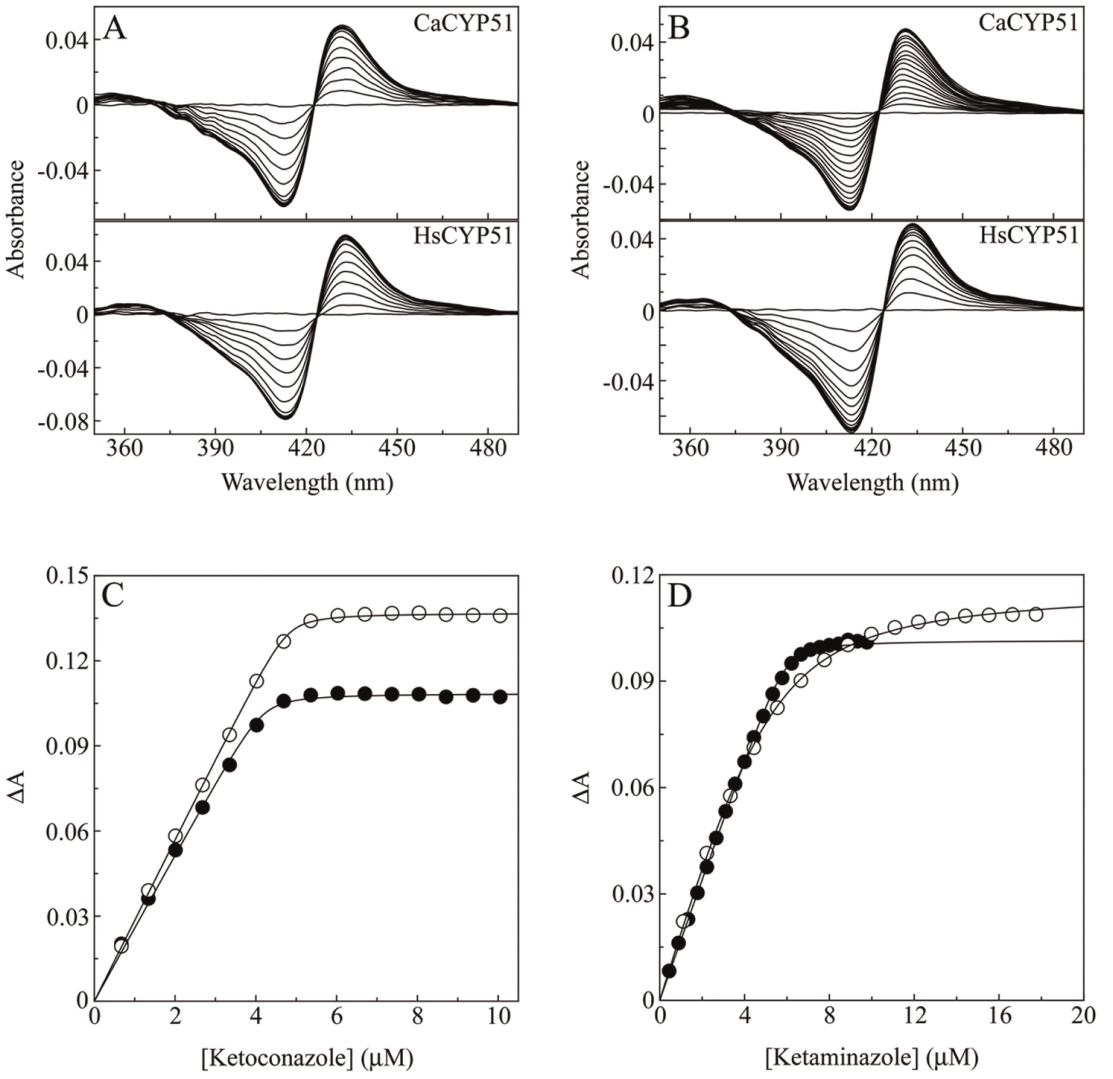
Binding properties of ketoconazole and ketaminazole with CaCYP51 and HsCYP51. Azole antifungals were progressively titrated against 5 µM CaCYP51 (filled circles) and 5 µM HsCYP51 (hollow circles). The resultant type II difference spectra are shown for ketoconazole (A) and ketaminazole (B). Saturation curves for ketoconazole (C) and ketaminazole (D) were constructed and a rearrangement of the Morrison equation (45) was used to fit the data. The data shown represent one replicate of the three performed.

In order to evaluate the concept of an improved anti-inflammatory effect combined with antifungal potency, we examined the selectivity of ketoconazole and ketaminazole (**16**) against the human and *C. albicans* CYP51 proteins, HsCYP51 and CaCYP51 respectively. Binding ketoconazole and ketaminazole (**16**) with both CaCYP51 and HsCYP51 produced strong type II difference spectra (Figure 6) signifying direct coordination as the sixth ligand of the heme prosthetic group of CYP51 [58], [59]. Ketoconazole and ketaminazole (**16**) both bound tightly to CaCYP51 with *K*_d_ values of 27±5 and 43±5 nM, respectively. Tight binding is observed when the *K*_d_ for the ligand is similar to or less than the concentration of CYP51 present [60]. The similar *K*_d_ values obtained for ketoconazole and ketaminazole (**16**) suggest both azoles would be equally effective as antifungal agents against wild-type CaCYP51. This is understandable since the CYP51 potency of this class of molecules is predominantly due to their azole moiety, which is quite distant from the phenylenediamine core of ketaminazole (**16**). This data also compares with *K*_d_ values of 10 to 50 nM previously obtained for clotrimazole, econazole, fluconazole, itraconazole, ketoconazole, miconazole and voriconazole with CaCYP51 [39]. Ketoconazole bound 17-fold more tightly to HsCYP51 (*K*_d_ = 42±16 nM) compared to ketaminazole (**16**) (*K*_d_ = 731±69 nM), in contrast to the 1.6-fold difference observed with CaCYP51, suggesting that ketaminazole would interfere less with the host HsCYP51 and possibly other human CYPs than ketoconazole, conferring a therapeutic advantage. These results compare well with the previously reported *K*_d_ values of <100 nM for clotrimazole, econazole and miconazole, ∼180 nM for ketoconazole and ∼70 µM for fluconazole with HsCYP51 [40].

**Figure 7:**
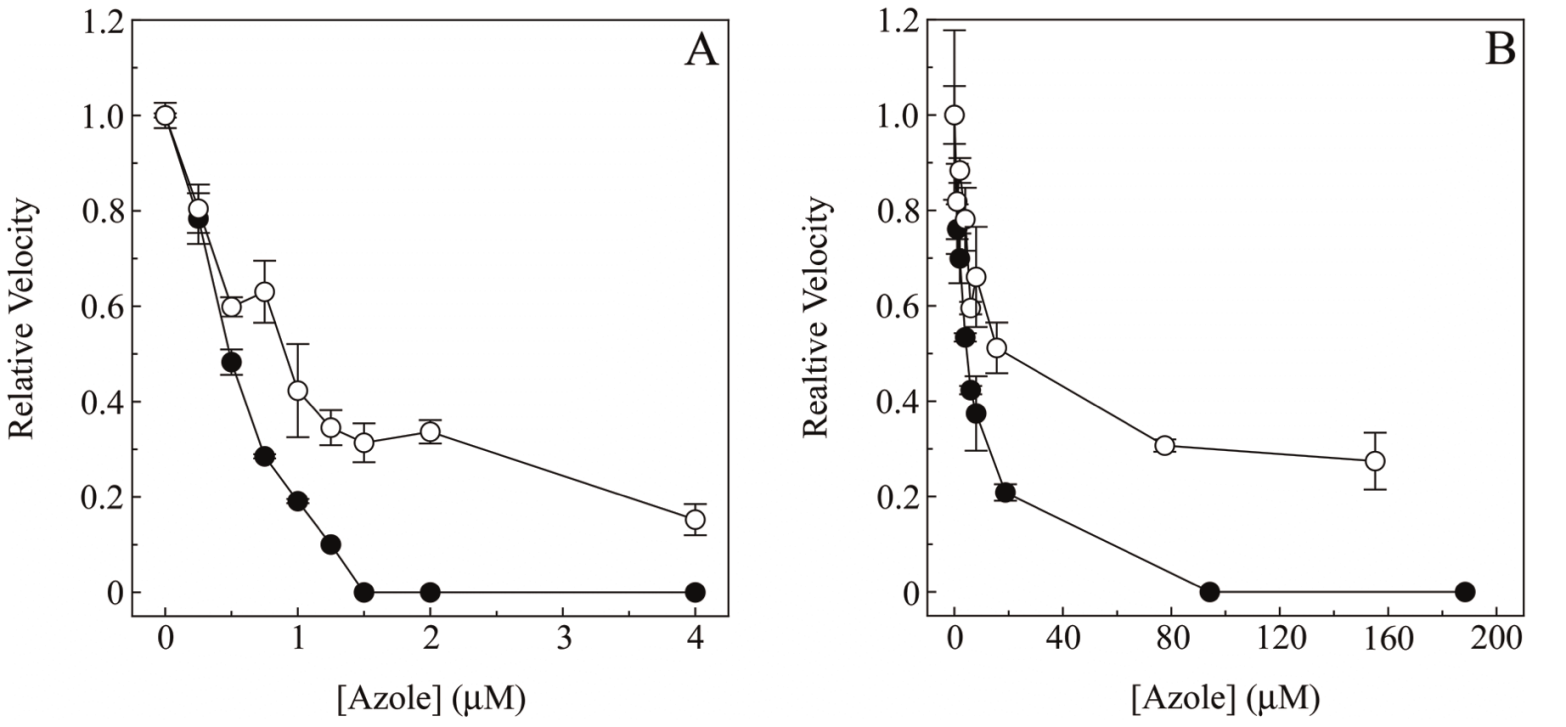
Determination of IC_50_ values for ketoconazole and ketaminazole with CaCYP51 and HsCYP51. CYP51 reconstitution assays (0.5-ml total volume) containing 1 µM CaCYP51 (A) or 0.3 µM HsCYP51 (B) were performed as detailed in Materials and Methods. Ketoconazole (solid circles) and ketaminazole (hollow circles) concentrations were varied from 0 to 4 µM for CaCYP51 and up to 190 µM for HsCYP51 with the DMSO concentration kept constant at 1% (vol/vol). Mean values from two replicates are shown along with associated standard deviation bars. Relative velocities of 1.0 were equivalent to 1.04 and 2.69 nmoles 14α-demetylated lanosterol produced per minute per nmole CYP51 (min^−1^) for CaCYP51 and HsCYP51, respectively.

The IC_50_ CYP51 reconstitution assay results (Figure 7) mirrored those of the azole binding results. CaCYP51 was strongly inhibited by both ketoconazole and ketaminazole (**16**) with IC_50_ values of ∼0.5 and ∼0.9 µM, respectively, confirming that both azoles bound tightly to CaCYP51. Interestingly, at 4 µM ketaminazole (**16**), CaCYP51 retained ∼15% CYP51 activity suggesting that lanosterol can displace ketaminazole (**16**) from CaCYP51 leading to ketaminazole (**16**) being a less effective inhibitor of fungal CYP51 enzymes *in vitro* than ketoconazole. HsCYP51 was less severely inhibited by both ketoconazole and ketaminazole (**16**) with IC_50_ values of ∼5 and ∼16 µM, respectively. This indicates that azole binding was less tight and suggested lanosterol can displace ketoconazole and especially ketaminazole (**16**) from HsCYP51. At 95 µM ketoconazole HsCYP51 was inactivated in contrast to the ∼30% CYP51 activity remaining in the presence of 155 µM ketaminazole (**16**). The 3-fold higher IC_50_ value of ketaminazole (**16**) over ketoconazole with HsCYP51 confirmed that ketaminazole (**16**) would be less disruptive to the CYP51 function of the host homolog than ketoconazole, conferring a therapeutic advantage for use as an antifungal agent. It should be noted that both itraconazole and posaconazole, both effective anti-fungal agents, could also have a phenylenediamine incorporated into their structures, thus conferring dual anti-fungal/anti-inflammatory properties on these therapeutics as well. We are currently investigating the properties of these modified anti-fungal agents further, with the hope of utilizing the phenylenediamine moiety as a simple modification for adding 5-LOX inhibitory potency to known therapeutics.

The fact that ketoconazole is both an anti-fungal and anti-inflammatory molecule is not a new phenomenon in the field of anti-fungal therapeutics. Previously, we determined that the common anti-fungal agent, chloroxine, was also a non-specific LOX inhibitor [61]. This fact suggested that the inherent selection process for the search for anti-seborrheic dermatitis agents could be responsible for the dual nature of the anti-fungal/anti-inflammatory therapeutics, such as chloroxine and ketoconazole. With this hypothesis in mind, the anti-fungal agent, ciclopirox (trade name Loprox), presented a structure that could be interpreted as a LOX inhibitor, with the *N*-hydroxyamide being a possible chelator. This was confirmed and ciclopirox was found to be both a potent inhibitor of 5-LOX (IC_50_ = 11±1 µM) and selective versus other AA processing enzymes (Figure 4). This dual nature of many anti-seborrheic dermatitis agents suggests that improving the 5-LOX potency of these therapeutics may be beneficial in their clinical efficacy.

## Conclusion

The current data indicate that the phenylenediamine chemotype reported herein is a potent inhibitor against 5-LOX, demonstrating enzyme selectivity and cellular activity. The mechanism of action is consistent with reduction of the active site ferric ion, similar to that seen for zileuton, the only FDA approved LOX inhibitor. It is interesting to note that unlike zileuton, which chelates the iron through the *N*-hydroxyurea, the phenylenediamine chemotype lacks an obvious chelating moiety, thus differentiating it from zileuton. Structural modification around the phenylenediamine core was well tolerated, however, even relatively minor changes to the phenylenediamine moiety resulted in a loss of activity, presumably due to changes in its reduction potential. This attribute was utilized to modify the structure of ketoconazole to include the phenylenediamine moiety and produce a novel inhibitor, ketaminazole (**16**). This novel compound demonstrated an *in vitro* 40-fold increase in potency against 5-LOX relative to ketoconazole. However, in whole blood ketaminazole demonstrated only a 2-fold greater potency than ketoconazole. In addition, the overall potency of ketaminazole was reduced by approximately 10-fold relative to its *in vitro* potency. It is currently unclear how the cellular environment is lowering the potency of ketaminazole, but pharmacokinetic investigations are currently underway to probe this further. Ketaminazole (**16**) had comparable potency against fungal CYP51 and improved selectivity against the human CYP51, relative to ketoconazole, which suggests a possible therapeutic advantage. This novel dual nature of ketaminazole (**16**), possessing both anti-fungal and anti-inflammatory activity, could potentially have therapeutic uses against fungal infections that have an anti-inflammatory component.

## Supporting Information

File S1 Supporting Information on Inhibitor Synthesis and Characterization.
(DOCX)
