# Supplementary material for: Discovery of a Novel Dual Fungal CYP51/Human 5-Lipoxygenase Inhibitor: Implications for Anti-Fungal Therapy
Source: PLoS One. 2013 Jun 24;8(6):e65928. doi: 10.1371/journal.pone.0065928 (PMC3691235; doi:10.1371/journal.pone.0065928)
Supplement: File S1 — Supporting Information on Inhibitor Synthesis and Characterization. (DOCX) [file pone.0065928.s001.docx]

**Supporting Information (File S1)**

Discovery of a novel dual fungal cyp51/human 5-lipoxygenase inhibitor: Implications for anti-fungal therapy

Eric K. Hoobler, ^1^ Ganesha Rai, ^2^ Andrew G. S. Warrilow, ^3^ Steven C. Perry,^1^ Christopher J. Smyrniotis,^1^ Ajit Jadhav, ^2^ Anton Simeonov,^2^ Josie E. Parker, ^3^ Diane E. Kelly, ^3^ David J. Maloney ^2*^ S. L. Kelly ^3*^and Theodore R. Holman^1*^

^†^ This work was supported by the National Institutes of Health (GM56062 (TRH)), the Welsh Government (SLK) and the ERDF, via the Beacon project (SLK).

^1^ Chemistry and Biochemistry Department, University of California, Santa Cruz, CA 95064 USA

^2^ NIH Chemical Genomics Center, National Center for Advancing Translational Sciences, 9800 Medical Center Drive, MSC 3370 Bethesda, MD 20892-3370 USA

^3^ Institute of Life Science, College of Medicine, Swansea University, Swansea, Wales, SA2 8PP, United Kingdom.

*Author to which all inquires should be addressed. For D.J.M.: [maloneyd@mail.nih.gov](mailto:maloneyd@mail.nih.gov). For S.L.K. s.l.kelly@swansea.ac.uk. For T.R.H: [holman@ucsc.edu.](mailto:tholman@chemistry.ucsc.edu.)

Running title: Discovery of a Novel Dual Fungal CYP51/5-LOX Inhibitor.

**Representative procedures and characterization of phenylenediamine inhibitors.** A mixture of tert-butyl 4-(4-aminophenyl)piperazine-1-carboxylate (1.94 g, 6.99 mmol, 1 eq) and 5-bromofuran-2-carbaldehyde (1.26 g, 7.34 mmol, 1.05 eq) in MeOH (35 mL) and CH_2_Cl_2_ (35 mL) was added sodium triacetoxyborohydride (4.45 g, 20.98 mmol, 3 eq). The reaction mixture was stirred at room temperature for 5 h. The product was extracted with ethyl acetate and the organic layer was subsequently washed with water, bicarbonate and brine. The crude product obtained after evaporation of the solvent was purified on a biotage flash system^®^ eluting with 40% ethyl acetate in hexanes (Yield 1.94 g, 62 %)

***t*-Butyl-4-(4-(((5-(4-isocyanophenyl)furan-2-yl)methyl)amino)phenyl)piperazine-1-carboxylate (1)**: To a mixture of t-butyl 4-(4-(((5-bromofuran-2-yl)methyl)amino)phenyl)piperazine-1-carboxylate (0.172 mmol, 0.075 g, 1 eq) and (4-cyanophenyl)boronic acid (0.206 mmol, 30 mg, 1.2 eq) in 1 mL DME was added Pd(PPh_3_)_4_ (5 mol %, 0.09 mmol, 10 mg) and a 2.0 M aqueous solution of Na_2_CO_3_ (0.52 mmol). The mixture was irradiated in a microwave reactor for 30 minutes at 150 ^o^C. The solvent was removed by blowing air and the crude product was dissolved in DMF, passed through a palladium scavenger cartridge and finally purified in preparative HPLC. LC-MS: rt (min) = 5.10 (8.0 min run); ^1^H NMR (400 MHz, DMSO-*d*_6_) δ 7.86 – 7.74 (m, 3H), 7.10 (d, *J* = 3.4 Hz, 1H), 6.78 – 6.69 (m, 2H), 6.62 – 6.53 (m, 2H), 6.44 – 6.38 (m, 1H), 5.72 (t, *J* = 6.2 Hz, 1H), 4.24 (d, *J* = 6.1 Hz, 2H), 3.47 – 3.34 (m, 4H), 2.81 (dd, *J* = 6.1, 4.1 Hz, 4H), 1.38 (d, *J* = 7.9 Hz, 9H); HRMS (ESI) *m*/*z* (M+H)+ calcd. for C_27_H_31_N_4_O_3_, 459.2391; found 459.2374.

***t*-Butyl-4-(4-(((5-(4-isocyanophenyl)furan-2-yl)methyl)amino)phenyl)piperidine-1-carboxylate (2) :** LC-MS: rt (min) = 6.54 (8.0 min run); ^1^H NMR (400 MHz, DMSO-*d*_6_) δ 7.80 (m, 2H), 7.10 (d, *J* = 3.3 Hz, 1H), 6.91 (d, *J* = 8.2 Hz, 2H), 6.57 (d, *J* = 8.2 Hz, 2H), 6.42 (d, *J* = 3.3 Hz, 2H), 5.97 (t, *J* = 6.2 Hz, 1H), 4.26 (d, *J* = 6.0 Hz, 2H), 3.28 (m, 1H), 2.46 (m, 4H), 1.67 – 1.58 (m, 4H), 1.36 (s, 9H); HRMS (ESI) *m*/*z* (M+H)+ calcd. for C_28_H_32_N_3_O_3_, 458.2438; found 458.2416.

***t*-Butyl-4-(6-(((5-(4-isocyanophenyl)furan-2-yl)methyl)amino)pyridin-3-yl)piperazine-1-carboxylate (6):** LC-MS: rt (min) = 4.886 (8.0 min run); ^1^H NMR (400 MHz, DMSO-*d*_6_) δ 7.81 (q, *J* = 8.4 Hz, 4H), 7.69 (d, *J* = 2.8 Hz, 1H), 7.22 (dd, *J* = 8.9, 2.9 Hz, 1H), 7.11 (d, *J* = 3.4 Hz, 1H), 6.62 (t, *J* = 6.0 Hz, 1H), 6.52 (d, *J* = 9.0 Hz, 1H), 6.37 (d, *J* = 3.4 Hz, 1H), 4.46 (d, *J* = 5.9 Hz, 2H), 3.41 (t, *J* = 5.2 Hz, 4H), 3.01 (s, 6H), 2.85 (t, *J* = 5.1 Hz, 4H), 1.47 (s, 1H), 1.39 (s, 9H), 1.22 (d, *J* = 5.7 Hz, 1H); ); HRMS (ESI) *m*/*z* (M+H)+ calcd. for C_26_H_30_N_5_O_3_, 460.2343; found 460.2343.

***t***-**Butyl-4-(4-(((2-(4-cyanophenyl)oxazol-5-yl)methyl)amino)phenyl)piperazine-1-carboxylate (8) :** LC-MS: rt (min) = 4.645 (8.0 min run);^1^H NMR (400 MHz, DMSO-*d*_6_) δ 8.09 – 8.01 (m, 2H), 8.00 – 7.92 (m, 2H), 7.22 (s, 1H), 6.79 – 6.70 (m, 2H), 6.64 – 6.56 (m, 2H), 5.77 (t, *J* = 6.2 Hz, 1H), 4.33 (d, *J* = 6.2 Hz, 2H), 3.38 (t, *J* = 5.0 Hz, 4H), 2.82 (t, *J* = 5.1 Hz, 4H), 1.37 (s, 9H); HRMS (ESI) *m*/*z* (M+H)+ calcd. for C_26_H_30_N_5_O_3_, 460.2343; found 460.2323.

**1-(4-(4-(((5-(4-bromophenyl)furan-2-yl)methyl)amino)phenyl)piperazin-1-yl)ethanone (12) :** LC-MS: rt (min) = 4.645 (8.0 min run);^1^H NMR (400 MHz, DMSO-*d*_6_) δ 8.09 – 8.01 (m, 2H), 8.00 – 7.92 (m, 2H), 7.22 (s, 1H), 6.79 – 6.70 (m, 2H), 6.64 – 6.56 (m, 2H), 5.77 (t, *J* = 6.2 Hz, 1H), 4.33 (d, *J* = 6.2 Hz, 2H), 3.38 (t, *J* = 5.0 Hz, 4H), 2.82 (t, *J* = 5.1 Hz, 4H), 1.37 (s, 9H); HRMS (ESI) *m*/*z* (M+H)+ calcd. for C_25_H_29_N_6_O_3_, 461.2296; found 461.2288.

**Synthesis of ketaminazole.** A mixture of 2-((1H-imidazol-1-yl)methyl)-2-(2,4-dichlorophenyl)-1,3-dioxolan-4-yl)methyl methanesulfonate (0.9 g, 2.21 mmol, 1eq) [purchased from Toronto Research Chemicals] and 1-(4-(4-aminophenyl)piperazin-1-yl)ethanone (0.581 g, 2.65 mmol, 1.2 eq) in a microwave vial was stirred neat at 130 ^o^C for 1 h. The crude product was dissolved in DMSO and purified ISCO^®^ reverse phase flash system using a water and acetonitrile solvent system. The product fractions were pooled and lyophilized to get a brown solid. LC-MS: rt (min) = 3.64 (8.0 min run); ^1^H NMR (400 MHz, DMSO-*d*_6_) δ 9.10 (t, *J* = 1.4 Hz, 1H), 7.72 (d, *J* = 2.1 Hz, 1H), 7.67 – 7.63 (m, 2H), 7.62 (s, 1H), 7.51 (dd, *J* = 8.5, 2.1 Hz, 1H), 7.11 – 6.94 (m, 2H), 6.67 – 6.49 (m, 2H), 4.89 – 4.72 (m, 2H), 4.23 – 4.07 (m, 1H), 3.91 – 3.41 (m, 6H), 3.27 – 2.79 (m, 6H) and 2.05 (s, 3H)^.^; HRMS (ESI) *m*/*z* (M+H)+ calcd. for C_26_H_30_Cl_2_N_5_O_3_, 530.1720; found 530.1698.
